# Musical Representation of Dendritic Spine Distribution: A New Exploratory Tool

**DOI:** 10.1007/s12021-013-9195-0

**Published:** 2014-01-07

**Authors:** Pablo Toharia, Juan Morales, Octavio de Juan, Isabel Fernaud, Angel Rodríguez, Javier DeFelipe

**Affiliations:** 1 Departamento de Arquitectura y Tecnología de Computadores y Ciencia de la Computacióne e Inteligencia Artificial, Universidad Rey Juan Carlos (URJC), Madrid, Spain; 2 Cajal Blue Brain Project, Universidad Politécnica de Madrid (UPM), Madrid, Spain; 3 Conservatorio Profesional Guitarrista José Tomás de Alicante, Alicante, Spain; 4 Laboratorio Cajal de Circuitos Corticales, Centro de Tecnología Biomédica, Universidad Politécnica de Madrid (UPM) and Instituto Cajal (CSIC), Madrid, Spain; 5 Department de Arquitectura y Tecnologíade Sistemas Informáticos, Universidad Politécnica de Madrid (UPM), Madrid, Spain

**Keywords:** Audio analysis, Cerebral cortex, Pyramidal cells, Dendritic spine morphology, Music translation

## Abstract

Dendritic spines are small protrusions along the dendrites of many types of neurons in the central nervous system and represent the major target of excitatory synapses. For this reason, numerous anatomical, physiological and computational studies have focused on these structures. In the cerebral cortex the most abundant and characteristic neuronal type are pyramidal cells (about 85 % of all neurons) and their dendritic spines are the main postsynaptic target of excitatory glutamatergic synapses. Thus, our understanding of the synaptic organization of the cerebral cortex largely depends on the knowledge regarding synaptic inputs to dendritic spines of pyramidal cells. Much of the structural data on dendritic spines produced by modern neuroscience involves the quantitative analysis of image stacks from light and electron microscopy, using standard statistical and mathematical tools and software developed to this end. Here, we present a new method with musical feedback for exploring dendritic spine morphology and distribution patterns in pyramidal neurons. We demonstrate that audio analysis of spiny dendrites with apparently similar morphology may “sound” quite different, revealing anatomical substrates that are not apparent from simple visual inspection. These morphological/music translations may serve as a guide for further mathematical analysis of the design of the pyramidal neurons and of spiny dendrites in general.

****Electronic Supplementary Material**:**

The online version of this article (doi:10.1007/s12021-013-9195-0) contains supplementary material, which is available to authorized users.

## Introduction

Dendritic spines (for simplicity, spines) are small protrusions along the dendrites of many types of neurons in the central nervous system. These structures were discovered and named by Cajal in 1888 (Cajal 1888) in the cerebellar cortex of the hen and later described in various regions of the nervous system of a variety of vertebrate species. They are particularly abundant in the cerebellar cortex, basal ganglia, and cerebral cortex, where they represent key elements in the microcircuits. In the cerebral cortex, the most abundant and characteristic neuronal type are pyramidal cells (about 85 % of all neurons) and their dendritic spines are the main postsynaptic target of excitatory glutamatergic synapses (DeFelipe and Fariñas 1992). Almost all dendritic spines establish at least one synapse (Arellano et al. 2007b) and therefore changes in the number of spines in the dendritic arbors of neurons may influence cortical functions at both the cellular and system level. The analysis of the pattern of dendritic spine distribution along the dendrites is also an important feature to understand how the synaptic inputs on a given dendrite are organized (O’Brien and Unwin 2006) and it has been proposed that a correlation exists between morphological and functional parameters of dendritic spines (Spruston 2008; Kasai et al. 2010; Yuste 2010).

Thus, dendritic spines are key elements for understanding brain cognition and memory in both healthy and diseased brain and for better understanding the synaptic organization of the cerebral cortex.

Much of the structural data on dendritic spines produced by modern neuroscience involves the quantitative analysis of image stacks from light and electron microscopy, using standard statistical and mathematical tools as well as specific software-based methods to quantify the number of spines, to determine their morphological parameters (volume, length and shape) and to analyze their spatial distribution. Here, we present a new method with musical feedback for exploring dendritic spine morphology and distribution patterns in pyramidal neurons. This method is based on the work of Morales et al. (2012), where it was shown that the study of the distribution of spines can be greatly improved by introducing the audio channel as a new source of information for exploring the samples.

In the study by Morales et al., the visual analysis performed using straightening and unrolling transforms was complemented with simple sound feedback. These transforms result in a planar, unfolded arrangement, reducing the data dimensionality from 3D to 2D, while preserving the most relevant spatial properties from the original distribution. The transforms were implemented in DISPINE, a new software tool that supports the interactive visual analysis of 3D patterns. The interaction process allows users to dynamically change the visualization parameters, allowing the representation of data in such a way that the presence of relevant information is maximized.

In the present study, the audio stimuli perceived by the user is greatly improved by synthesizing music. The relevant morphological features of the spine structure and distribution being analyzed are transformed to musical notes. Thus, reproduction of the musical phrases obtained provides a very quick and easy way to discriminate between the different scenarios being studied. To test the methodology developed in the present article, we used previously published data from our laboratory (Benavides-Piccione et al. 2012) obtained from individual spines that were completely 3D reconstructed along the length of apical and basal dendrites of layer III pyramidal neurons in the cingulate cortex of two male humans (aged 40 and 85), using intracellular injections of Lucifer yellow in fixed tissue.

## Materials and Methods

### Tissue Preparation

The material used in the present study was prepared and analyzed in a previous study (Benavides-Piccione et al. 2012). Briefly, brain samples were obtained from two human males (aged 40 and 85). This tissue (kindly supplied by Dr. I. Ferrer, Instituto de Neuropatología Servicio de Anatomía Patológica, IDIBELL - Hospital Universitario de Bellvitge, Barcelona, Spain) was obtained at autopsy (2–3 h. post-mortem). The brains were immediately immersed in cold 4 % paraformaldehyde in 0.1 M phosphate buffer, pH 7.4 (PB) and sectioned into 1.5-cm-thick coronal slices. Small blocks of cortex ( 15 × 10 × 10 mm) were then transferred to a second solution of 4 % paraformaldehyde in PB for 24 h. at 4^∘^C. The tissue used in the present study was from the anterior cingulate gyri (Brodmann’s area 24; (Garey 1994)).

### Intracellular Injections

Coronal sections (250 *μ*m) were obtained with a Vibratome and labelled with 4,6 diamino-2-phenylindole (DAPI; Sigma, St Louis, MO) to identify cell bodies. Pyramidal cells were then individually injected with Lucifer Yellow (LY; 8 % in 0.1 M Tris buffer, pH 7.4), in cytoarchitectonically identified layer III of the anterior cingulate gyrus. LY was applied to each injected cell by continuous current until the distal tips of each cell fluoresced brightly, indicating that the dendrites were completely filled and ensuring that the fluorescence did not diminish at a distance from the soma (for a detailed methodology of the cell injections, see Elston and Rosa (1997), Elston et al. (2001), Ballesteros-Yáñez et al. (2010)).

### Immunocytochemistry

**Fig. 1:**
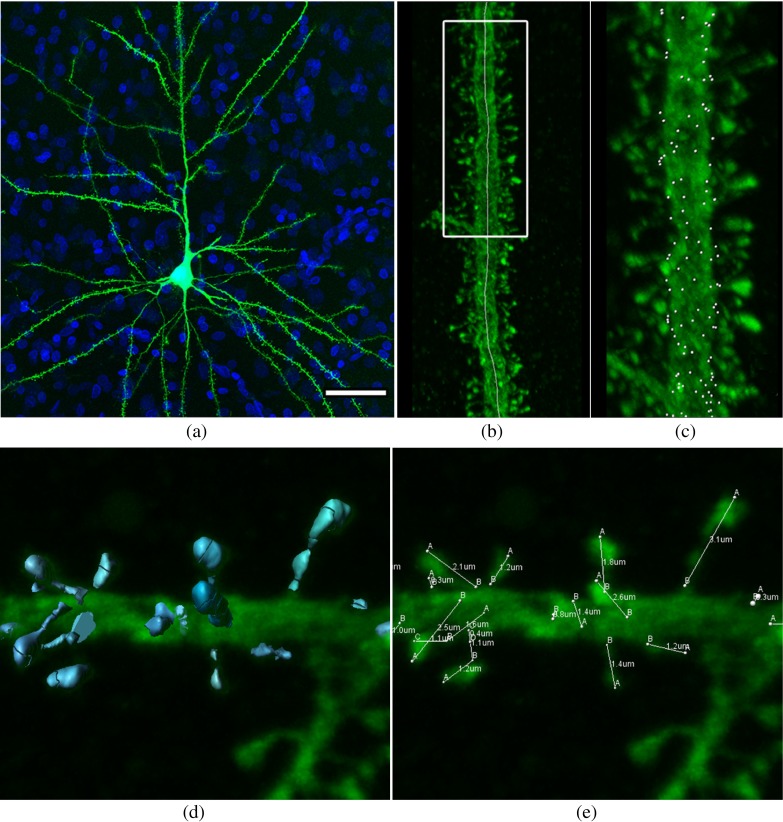
**a** Confocal microscopy image of an intracellularly injected layer III pyramidal neuron of the human cingulate cortex (image taken from Benavides-Piccione et al. (2012)). **b** High magnification image showing the apical dendritic segment analyzed for the 40 year old case, with the longitudinal axis traced through the middle of the dendritic shaft. **c** Detail of the same dendritic segment including spines and their points of insertions (*white dots*) in the dendritic shaft. **d** Confocal microscopy image showing a labeled dendritic segment with spines of different sizes and lengths. To three-dimensionally reconstruct the complete morphology of each spine, a particular threshold was selected to constitute a solid surface that exactly matched the contour of that spine. **e** For each individual spine, its length was manually marked from its point of insertion in the dendritic shaft to the distal tip of the spine, while rotating the image in three dimensions. Scale bar (in **a**): **a** 40 *μ*m; **b** 8 *μ*m; **c** 4.5 *μ*m; **d**–**e** 2 *μ*m

Following the intracellular injection of pyramidal neurons (Fig. 1a), sections were stained for LY using rabbit antisera against LY (1:400,000; generated at the Cajal Institute) diluted in stock solution (2 % bovine serum albumin, 1 % Triton X-100, 5 % sucrose in PB). The sections were then incubated in biotinylated donkey anti-rabbit IgG (1:100; Amersham, Buckinghamshire, UK) and Alexa fluor 488 conjugated streptavidin (1:1000; Molecular Probes). Finally, sections were mounted in 50 % glycerol in PB.

### Imaging and Quantitative Analysis

All the images for this study had already been analyzed as part of a previous study from our laboratory (Benavides-Piccione et al. 2012). Briefly, sections were imaged with a Leica TCS 4D confocal scanning laser attached to a Leitz DMIRB fluorescence microscope. Fluorescent labelling profiles were imaged, using an excitation wavelength of 491 nm to visualize Alexa fluor 488. Segments of the main apical dendrite (100 *μ*m) and complete basal dendrites of each case, were scanned at high magnification (63 × glycerol, z-step of 0.28 *μ*m) (see examples in Fig. 1b, c). For each stack of images, confocal parameters were set so that the fluorescence signal was as bright as possible while ensuring that there were no saturated pixels within the spines.

Spine structure was analyzed using Imaris 7.2.1 (Bitplane AG, Zurich, Switzerland), as described in Benavides-Piccione et al. (2012). We then reconstructed the complete morphology of each dendritic spine.

The analysis used here was taken from data partially presented in Benavides-Piccione et al. (2012). Briefly, the 100 *μ*m long segments of apical dendrites or the complete basal dendrites were analyzed every 10 *μ*m of dendritic length and an axis was manually traced through the middle of the dendritic shaft (Fig. 1b, c). Regarding the dendritic spines, the following parameters were used:

Spine volume.: For each individual dendritic spine, a particular intensity threshold surface was selected among a range of 7 to 10 different thresholds to constitute a solid surface that exactly matched the contour of each dendritic spine (Fig. 1d). In some cases, a single spine was reconstructed by combining several surfaces of different intensity thresholds.
Spine length: was manually marked in each individual dendritic spine from its point of insertion in the dendritic shaft to the distal tip of the spine (Fig. 1e).These spines’ data and points of insertion were used as the markers for the audio analysis presented here (Fig. 1c–e).

## Multimodal Analysis

Analysis of spine distribution patterns is a very difficult task due to the 3D irregular arrangement of the reference markers, the large number of structures being studied and the dendrite’s geometry (Fig. 1). In order to improve the analysis process, users can mix raw data together with multimodal information from visual and audio channels. For example, the user may display visual references such as the position of the spines’ insertion points, the dendrites’ medial axis, a 3D curve interpolating the spines’ insertion points, the straightened spines’ distribution or the result of the unrolling transformation. Additionally, visual mapping is performed using colour oriented ellipsoid glyphs, as described in Morales et al. (2012).

In the present work, multimodal analysis including musical feedback is used. This approach makes use of the humans’ ability to recognize audible frequencies and rhythms (temporal repetition patterns of audible frequencies; (Mansur et al. 1985)). Thus, synchronized with the visual information, the user can listen to musical notes of the features mapped in the audio channel. Another possibility would be to consider only one of the available channels, visual or audio, selecting the more appropriate option according to the data under analysis.

For spines, morphological features of interest are the spine’s volume, length, orientation and the spatial position along the dendrite. Regarding the spatial position, parameters of interest are the spines’ angular position around the dendrites’ medial axis and the relative positions along the medial axis in the original data sequences. Several references are available for fixing the spatial position. For example, the distal tip, the insertion point or the centroid of the spines. In this work the insertion point has been selected because it is more stable than the other options (Portera-Cailliau et al. 2003). Throughout the paper, the word “marker” will be synonymous with the term “spine marker”, or simply “spine”.

In the following paragraphs some background information about the properties of music is first introduced, and then, the audio mapping for transforming the morphological features of the spines’ and their distributions under study to musical sounds is described.

### Musical Background

The following attributes define how humans perceive musical sounds (Sadie and Grove 1995a):

**Timbre**: (also known as color or tone quality): This is the property that allows us to distinguish between different sources of sounds, like voices (tenor, soprano, bass, mezzo-soprano, …) or musical instruments (piano, violin, flute, …) because of the different distributions of their harmonics ( Play examples).
**Pitch:** this attribute allows us to order the sounds according to a scale of reference. This quality is related to the frequency of the sound wave. Bass voices produce sounds with lower pitch than the higher pitch of soprano voices ( Play examples).
**Amplitude:** this property refers to the volume or loudness, with the magnitude reflecting the strength of the sounds ( Play examples).
**Duration:** interval of time or length of a given sound.

**Fig. 2:**
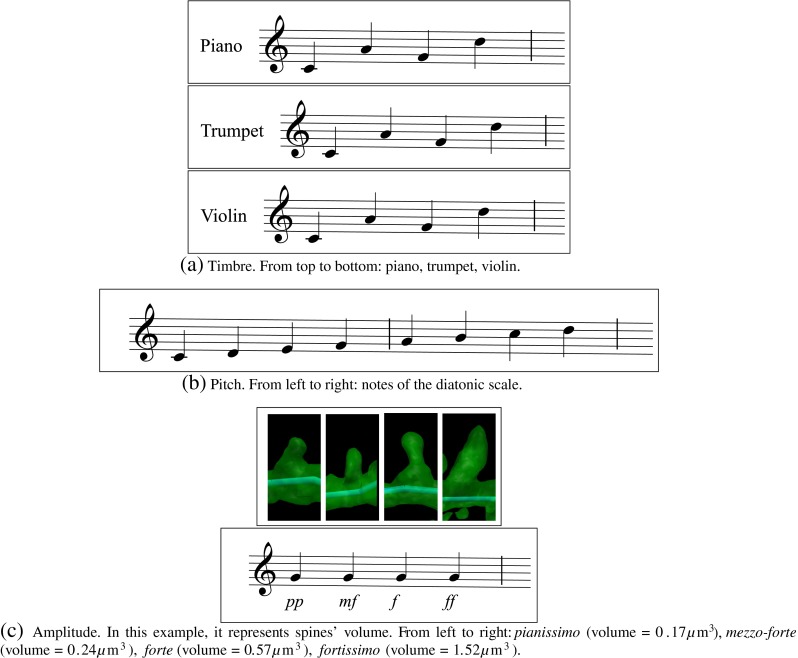
Basic attributes of music: timbre, pitch and amplitude. Notice that the dendrite’s medial axis (light blue) is also shown in all the images as a common visual reference (diameter=0.20 μm). Click on images to hear the sound

**Fig. 3:**
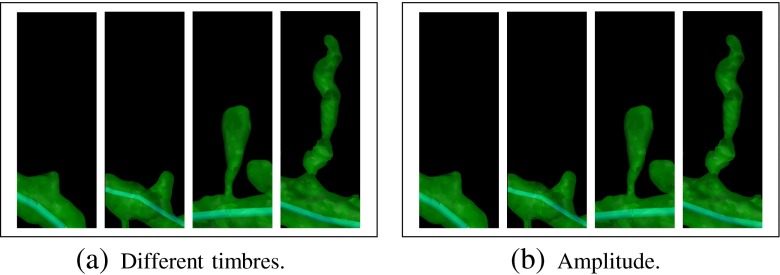
Two alternative musical mappings for the spines’ length. In (**a**), from left to right: violin’s pizzicato (length = 0.35 μm), piano (length = 1.21 μm, trumpet (length = 2.28 μm), violin (length = 4.29 μm). In (**b**), from left to right: *pianissimo* (length = 0.35 μm), *mezzo-forte* (length = 1.21 μm), *forte* (length = 2.28 μm), *fortissimo* (length = 4.29 μm). Click on the image to hear the sound

1 One way to formalize the representation of these attributes is to annotate music using staves (Sadie and Grove 1995b). Figure 2 shows several examples of these attributes. Figure 3a shows several examples of the timbres selected.

### Musical Mapping

The piano has been selected as the basic timbre for playing sounds. This instrument has several properties that perfectly suit the purposes of this work (Jourdain 2001). Perhaps due to the development in the quality of keyboards as a substitute for original pianos, or due to piano’s percussive nature, the fact was that, after testing many instruments (string, wind, percussion instruments or voices) with different software for audio, the closest to the natural sound was the piano. This point is considered to be of great importance to encourage listener attention and improve their overall listening experience.

In addition, from the programming point of view, it is also very simple to implement.

The duration of the notes has been fixed to a constant value because the ability of human beings to distinguish between similar lengths of sounds is very limited. This limitation would make it difficult to notice the characteristics of two consecutive spines in an area that is densely populated with spines.

**Fig. 4:**
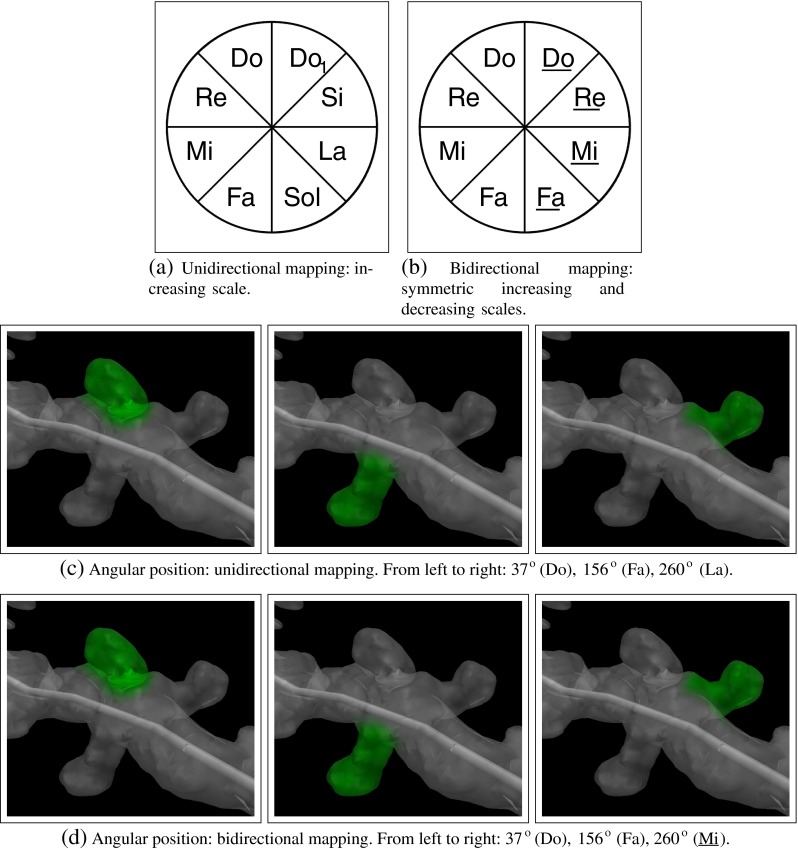
Two alternative mappings of the angular position: unidirectional and bidirectional. With unidirectional mapping, all the sounds are listened from all the loudspeakers available. With bidirectional mapping, stereophonic sound properties are applied and the underlined notes are listened from the “*right*” loudspeaker, while the non-underlined ones are listened from the “*left*” loudspeaker. Please, click on the images to hear the sound

**Fig. 5:**
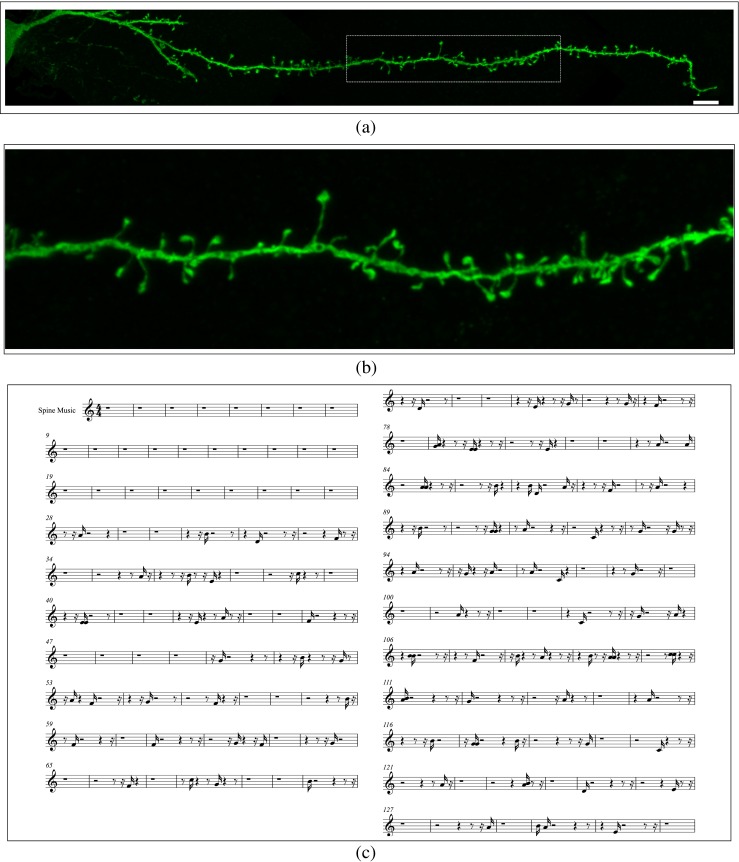
**a**, **b** Confocal microscopy image of a completely reconstructed basal dendrite (starting at the soma and continuing to the distal tip of the dendrite) of an intracellularly injected layer III pyramidal neuron of the human cingulate (from and 85 year old male). The reconstruction was made out of three consecutive confocal microscopy stacks of images following the dendrite axis. **c** Music sheet. Scale bar (in **a**): **a** 8 *μ*m; **b** Zoom × 3.4 over the boxed area in (**a**). In (**a**), the displacement along the dendrite begins from the soma giving a basal sound initially until the first spine is reached (musical notes). Click on images (**a**) and (**b**) to hear the sound
  This mapping has been chosen by taking into account the fact that humans perceive a note of a given length as being shorter or longer, depending on which instrument is used to produce it. We considered four instruments and, for the sake of comparison that follows, we assume each instrument produces a sound of the same length. This sound (of a given length) will be perceived as short when produced by the violin pizzicato and piano, which are both percussive instruments. Therefore, shortest spines are represented by pizzicatos because the subjective length of the note is shorter than the piano. The trumpet and violin are at the other end of the spectrum, and have been chosen to represent longer spines since the subjective perception is that the length of the note is larger. In the case of the violin, the subjective length of the note is the largest of the four instruments, and therefore, this timbre has been assigned to the group comprising the longest spines.
  However, when only length is enabled, the mapping applied in this case is the same as the one described for the volume feature with the following intervals (Fig. 3b):
  1. < 1*μ*m → **pp**(*pianissimo*/very soft)
  2. 1 − 2*μ*m → **mf**(*mezzo-forte*/moderately loud)
  3. 2 − 3*μ*m → **f** (*forte*/loud)
  4. > 3*μ*m → **ff** (*fortissimo*/very loud)

Musical mapping has been performed using the following transformations of morphological features into musical parameters:

1. **Spatial position** along the dendrite: Activation of sounds according to the spatial arrangement of spines along the dendrites’ medial axis, resulting in audible musical rhythm. The reproduction of sound implies the concept of the sequence of notes. Since users would be confused if the sound activation were not mapped to any sequenced parameter, mapping the sound activation to the spatial position is the most obvious and appropriate choice.
2. **Angular position:** The pitch has been chosen for mapping the angular position of the spines’ around the dendrites’ medial axis. As pitch is a discrete magnitude and angular position is continuous, the possible range of angles [0, 360^∘^] is separated into several “wedges”, assigning one single pitch value to each wedge, one of the pieces obtained after the discretization. To link the original range of angular values to the pitch values selected, the following two alternatives can be applied:
  Unidirectional mapping (Fig. 4a): The pitch is gradually increased from the minimum ( 0^∘^) to the maximum ( 360^∘^) ( Play examples). This alternative allows us to print the usual musical score representation, which gives neuroscientists an additional visual way to distinguish and classify the different distributions according to the features of the combination of different parameters examined (see section “Musical Background”):
    - A diatonic scale was chosen because this correlation is an accepted standard. It would be better for the researcher to listen to the most familiar occidental (western) scale rather than other ones, like the chromatic or the one-tone scales; using a diatonic scale would allow easier identification and memorization of tones.
    - Subdividing the 360^∘^range of the angular position into 4, 7 or 14 pieces has been made possible to facilitate more comfortable “cutting” of the dendrite’s axial surface since the most suitable option can be chosen according to specific analysis. Obviously the greater the number of “wedges”, the greater the spatial accuracy achieved.
    - A similar case occurs in the “auto-selection” of the scanning step rate according to the temporal length of the recording. The slower the speed of the scanning step, the clearer it is to the listener (avoiding simultaneous sound, the chords). However, slow speeds also lead to a longer overall duration, which is less engaging for the listener.
    - If the corresponding recordings of two or more dendritic spines matched in terms of the spine size and scanning step speed, the selection of unidirectional mapping also allows us to synthesize compositions with several instruments, like a trio or quartet. That is to say, the acoustical and visual representation of images may be synchronized from different individuals (even animals) in order to compose their characteristic physiological features, which could provide us with a holistic vision of the analysis.
  Bidirectional mapping is the second alternative approach (Fig. 4b): The pitch is gradually increased from a minimum (denoting 0^∘^) to the half of the angular maximum (denoting 180^∘^). Beyond 180^∘^, it then progressively decreases, in a symmetrical manner until it reaches the minimum pitch level again at 360^∘^. Although the same notes are used in both halves, they can be distinguished using the properties of the stereophonic sounds: the same note is projected from either the left or right loudspeaker depending on the angular position of the spine. For example, according to the discretization applied in Fig. 4 (8 intervals of 45^∘^), a value of 15^∘^ is represented by a Do heard from the left loudspeaker, while a value of 345^o^ is also represented by a Do but in this case from the right loudspeaker ( Play examples).
  Figures 4c and d show several examples where the unidirectional and the bidirectional mappings are applied.
3. **Spines’ volume:** The sound’s amplitude has been selected for mapping the spine volume. It seems only natural to represent the magnitude of the spines’ volume by directly linking it to the intensity of the notes: higher spine volume values will produce louder sounds, while the sounds produced by smaller spines will be softer ( Play examples). Four groups have been established based on the distribution frequency of the spines volumes (Benavides-Piccione et al. 2012). Each category has been assigned to a different volume of the white tone (pure tone):
  < 0. 20*μ*m ^3^ → **pp**(*pianissimo*/very soft)
  0. 20 − 0. 40*μ*m ^3^ → **mf**(*mezzo-forte*/moderately loud)
  0. 40 − 0. 60*μ*m ^3^ → **f** (*forte*/loud)
  > 0. 60*μ*m ^3^ → **ff** (*fortissimo*/very loud)
4. **Spines’ length:** When both length and volume features are enabled, like in Fig. 5 the timbres mentioned above have been applied to the four categories defined for the length (Fig. 3a). Each category has been assigned to a different timbre:
  1. < 1*μ*m → violin’s pizzicato
  2. 1 − 2*μ*m → piano
  3. 2 − 3*μ*m → trumpet
  4. > 3*μ*m → violin

The researchers can choose, combine and adapt their preferences with regard to the combinations of the morphological parameters being studied and the corresponding musical parameters selected for the multimodal analysis. This customization allows the users to become more efficient and automatic in their day-to-day work. For example, users can disable angle mapping if they only want to focus on volume and/or length mapping. In this case, pitch can be used to map the volume of the spines instead of listening to different timbres as occurs in the default operation.

Figures 2, 3 and 4 show several examples illustrating the musical mapping performed with the original spines and the corresponding sounds produced. Figure 5 shows the result of applying these concepts to a basal dendrite of an intracellularly injected layer III pyramidal neuron of the human cingulate cortex.

## Discussion

The tool we have created is not intended to replace the standard morphometric analysis of spines or the figures that illustrate these structures. It should be considered as an additional tool that helps the user to compare between dendrites and explore several features of the spines and to discover new possible patterns.

**Fig. 6:**
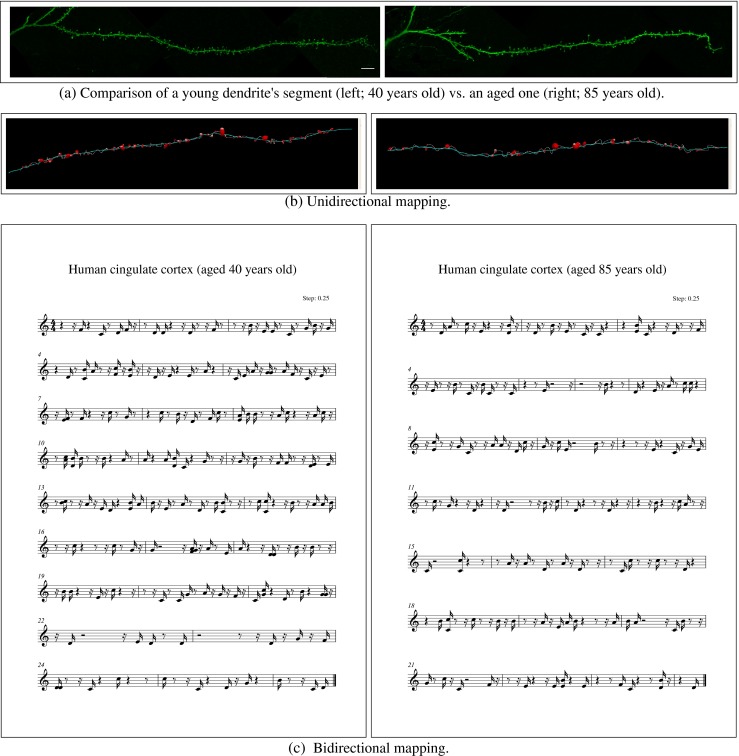
Confocal microscopy images showing part of the labeled dendritic segments analyzed in this study (young vs. aged (a)), the symbolic representations obtained with the visual mapping applied (b), and the musical scores obtained for the younger (left column) and the aged case (right column). In these examples, only angular position and volume are displayed. Click on images to hear the sound. In the young case the dendritic segment displayed a total of 146 spines, whereas in the old case there were 97 spines, and they also differed in the morphological parameters of the spines (see text for details). Note that it is difficult to perceive these morphological differences by visual inspection. However, the morphological/music translation allows us to discern these differences. Scale bar (in (a)): 8 μm

Figure 6 shows an example of the usefulness of sound translation of the spines’ morphology and their distribution to compare two different dendritic segments. These confocal images were taken from layer III pyramidal neurons of the human cingulate cortex of two male cases aged 40 (Fig. 6a, left) and 85 (Fig. 6a, right). The dendritic segment corresponding to the younger case displayed a total of 146 spines (density, 1.41 spines/ *μ*m; average length of spines, 1.37 *μ*m; average volume of spines, 0.32 *μ*m ^3^), whereas in the older case there were 97 spines (density,1.03 spines/ *μ*m; average length of spines, 1.56 *μ*m; average volume of spines, 0.47 *μ*m ^3^). In spite of these differences, it is difficult to perceive these morphological differences by visual inspection. However, audio analysis reveals clearly that these dendritic segments “sound” quite different.

Another interesting application is the use of the tool to count spines and look for rhythms. For example, if the user turns off the controls in the web tool and plays the music, each note represents a spine and the resulting melody is equivalent to counting spines. As a result, it will be clear that, in the 85-year-old case, the distribution of spines seems to be clustered and that these clusters are repeated in some portions of the dendrite. Also, if the user looks for the distribution of large spines, for example, it will be immediately evident that trumpets often sound together suggesting that long spines tend to be clustered. These observations would have been less obvious with visual analysis alone. The next step would be to investigate the spatial design of these spines with mathematical tools.

In the current study, we show that data analysis of spines’ morphology and their distributions can be effectively performed using musical feedback that complements and greatly improves visual information. In addition, sound information has already been used in the field of neuroscience, mainly for studying brain activity but also for generating sounds based on neuronal activity. In the first case, several examples can be found over the last 15 years, with most of them focusing on analyzing electroencephalography (Jovanov et al. 1999; Hinterberger and Baier 2005; Vialatte et al. 2010; Mindy and Chang 2010; Leslie and Mullen 2011; Wu et al. 2011), and fMRI data (Thompson et al. 2009). In the second case, the generation of sounds has been applied to different tasks, such as complementing the visual information representing complex datasets of neural activity through hands-on interaction (Weinberg and Thatcher 2006) or for reproducing the neural activity given by a sequence of electrical pulses (spikes) according to the excitatory or inhibitory action of the synapses in the SANTIAGO system (Kerlleñevich et al. 2011). In this system, only audio information is perceived by users. In the present study, we made use of the ability of humans to easily perceive audible frequencies and rhythms (temporal repetition patterns of audible frequencies (Mansur et al. 1985)). This feedback has proved particularly useful when neuroscientists are trying to detect the presence of periodic patterns hidden within the data being analyzed. In our previous work (Morales et al. 2012), we illustrated the advantages of this method comparing two artificial distribution patterns of spines: helical and purely random distribution patterns. The helical pattern was tested because it has been proposed that the spines of dendrites of the fish and mammalian Purkinje cells (O’Brien and Unwin 2006) display a helical pattern of distribution. These authors hypothesized that this organization would maximize the opportunity of different spines to interact with different axons. Thus, we compared these two patterns of distribution of spines in pyramidal cells and it was clearly shown visually (by straightening and unrolling transformations to move the 3D analysis process to a planar, unfolded arrangement) and after sonification that the distribution of spines of pyramidal neurons is close to random, ruling out the hypothesis of a more structured format (Morales et al. 2012).

In the present study, by implementing multimodal analysis through the synchronization of music with visual information, the user can listen to musical notes of the features mapped in the audio channel facilitating the pattern analysis of spines’ distributions. Furthermore, we introduced the possibility of testing only one of the available channels, visual or audio, allowing the selection of the more adequate option for the particular data being analyzed. Particular emphasis was placed on analysis of the spine morphology, since this parameter reflects the functional characteristics of the spine. For example, the spine head volume is correlated with the area of the postsynaptic density, the number of postsynaptic receptors and the readily-releasable pool of transmitter (Harris and Stevens 1989; Nusser et al. 1998; Schikorski and Stevens 1999; Arellano et al. 2007a), whereas the length of the spine neck is proportional to the degree of biochemical and electrical isolation of the spine from its parent dendrite (Majewska et al. 2000a, b; Araya et al. 2006).

Furthermore, larger spines can generate greater synaptic currents than smaller spines (Matsuzaki et al. 2004) and it has been proposed that small spines are preferential sites for long-term potentiation induction, whereas large spines might represent physical traces of long-term memory (Matsuzaki et al. 2004; Kasai et al. 2010).

Thus, the study of the morphology of spines is crucial from the functional point of view. In addition, we aimed to analyze other morphological features of interest, such as the orientation and the spatial position of spines along the dendrite, in order to unravel the structural design of the spiny dendrites. For this reason, we developed the tool described here in order to map different musical attributes to the morphological features of the spines, including spine’s volume, length, orientation and the spatial position along the dendrite. In this way it was easy to analyze each of these morphological attributes by listening to musical notes of the features mapped in the audio channel. In the example we used in the present study to test the usefulness of the method by comparing dendritic segments of layer III pyramidal neurons in the cingulate cortex of two male humans (aged 40 and 85 years old), we showed that in spite of the statistical differences described by Benavides-Piccione et al. (2012), it was difficult to perceive these morphological differences by visual inspection. However, audio analysis reveals clearly that these dendritic segments “sound” quite different. Clearly this has implications given that changes in the number and morphology of spines have been related to cognition and memory and these morphological parameters are deeply affected in several brain diseases such as epilepsy, Alzheimer and schizophrenia, and under a variety of experimental conditions such as drugs of abuse, sensory deprivation, etc. Furthermore, these changes are only revealed after statistical analysis since it is often difficult, if not impossible, to detect these changes simply by visual inspection. Considering all of these points, we propose that the present exploratory methodology is an excellent tool to reveal hidden principles of anatomical organization. These morphological/music translations may serve as a guide for further mathematical analysis of the design of pyramidal neurons and of spiny dendrites in general.

## Information Sharing Statement

The dataset shown in the paper can be downloaded from http://cajalbbp.cesvima.upm.es/audispine.

The main goal of the website is to show the potential of this new exploratory tool. The version implemented in the website is not intended to be a fully functional software application for analyzing datasets. We are currently developing a software tool that includes the level of functionality and interactivity required for a software application designed for worldwide distribution. The website version implements feature mapping selection and common interaction issues like fast fix position by clicking with the left mouse button at any time during the reproduction in both views (dendrite view and audio cursor); selection of the region of interest brushing with the left mouse button in the dendrite view; and reproduction of data with several speeds.

All the audio tracks included in this study will also be available at the same url, together with additional multimedia material.

Recommended navigators to avoid compatibility issues: firefox, chrome or safari.

Recommended loudspeakers: stereo; minimum response frequency: 50–20,000 Hz; power output: > 1*W*.

## Electronic supplementary material

Below is the link to the electronic supplementary material.

(PDF 14.7 MB)

## Footnotes

1 If you experience difficulties reproducing the embedded multimedia material, please note that many PDF readers provide the option of saving these examples by clicking either with the right button of the mouse (with AdobeAcrobat Reader, evince, foxit, okular and xfig-pdf-viewer) or with the left button (with sumatra). The sound clip can then be reproduced using a multimedia file player (assuming that the required PCM codec is installed). Some PDF viewers have security options enabled by default. Please check these options if you cannot play the multimedia content with the viewers listed above.
